# Supplementary material for: Venomix: a simple bioinformatic pipeline for identifying and characterizing toxin gene candidates from transcriptomic data
Source: PeerJ. 2018 Jul 31;6:e5361. doi: 10.7717/peerj.5361 (PMC6074769; doi:10.7717/peerj.5361)
Supplement: Supplemental Information 6 [file peerj-06-5361-s006.gz › FinalOutput_1E-6/Phospholipase_A1_19/finaltree.pdf]

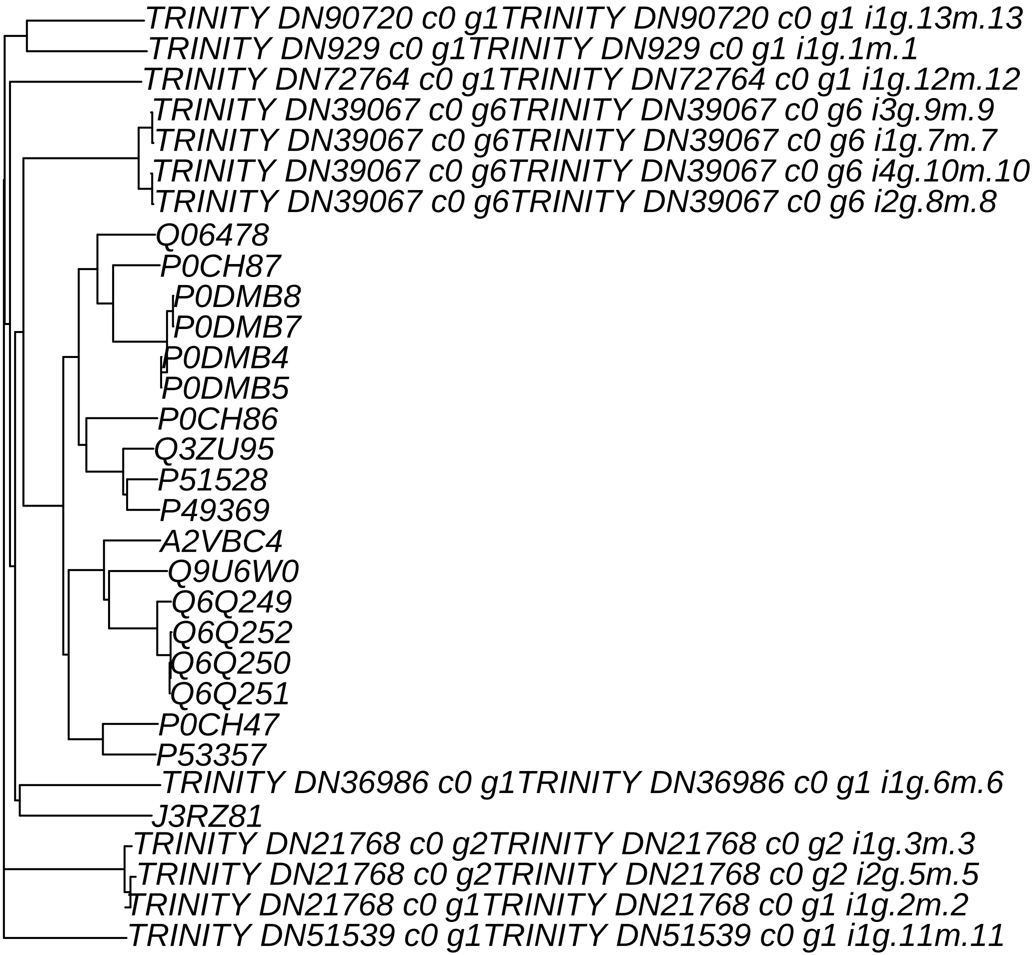
TRINITY DN90720 c0 g1TRINITY DN90720 c0 g1 i1g.13m.13
TRINITY DN929 c0 g1TRINITY DN929 c0 g1 i1g.1m.1
TRINITY DN72764 c0 g1TRINITY DN72764 c0 g1 i1g.12m.12
TRINITY DN39067 c0 g6TRINITY DN39067 c0 g6 i3g.9m.9
TRINITY DN39067 c0 g6TRINITY DN39067 c0 g6 i1g.7m.7
TRINITY DN39067 c0 g6TRINITY DN39067 c0 g6 i4g.10m.10
TRINITY DN39067 c0 g6TRINITY DN39067 c0 g6 i2g.8m.8
Q06478
P0CH87
P0DMB8
P0DMB7
P0DMB4
P0DMB5
P0CH86
Q3ZU95
P51528
P49369
A2VBC4
Q9U6W0
Q6Q249
Q6Q252
Q6Q250
Q6Q251
P0CH47
P53357
TRINITY DN36986 c0 g1TRINITY DN36986 c0 g1 i1g.6m.6
J3RZ81
TRINITY DN21768 c0 g2TRINITY DN21768 c0 g2 i1g.3m.3
TRINITY DN21768 c0 g2TRINITY DN21768 c0 g2 i2g.5m.5
TRINITY DN21768 c0 g1TRINITY DN21768 c0 g1 i1g.2m.2
TRINITY DN51539 c0 g1TRINITY DN51539 c0 g1 i1g.11m.11
